# Establish a new parameter “horizontal view-axial angle” and explore its role in the treatment of atlantoaxial instability diseases

**DOI:** 10.3389/fsurg.2022.947462

**Published:** 2023-01-06

**Authors:** Hongxiang Huang, Minfeng Sheng, Guangliang Zeng, Chao Sun, Rujun Li

**Affiliations:** ^1^ Department of Neurosurgery, Hainan General Hospital, Haikou, China; ^2^ Department of Neurosurgery, The Second Affiliated Hospital of Soochow University, Suzhou, China; ^3^ Department of Neurosurgery, Changshu No. 2 Peoples’ Hospital, Changshu, China

**Keywords:** atlantoaxial instability, atlantoaxial dislocation, basilar invagination, craniocervical junction, sagittal balance

## Abstract

**Objective:**

The objective of the study is to establish a new parameter that can be clearly measured on x-ray images to complement the description of the sagittal alignment of the craniocervical junction. The authors anticipate that this new parameter will enhance surgeons' understanding of the sagittal alignment of the craniocervical junction and play a positive role in the guidance of intraoperative reduction and in the evaluation of postoperative outcomes of patients with atlantoaxial instability.

**Methods:**

From November 2018 to June 2020, a total of 159 asymptomatic subjects who underwent frontal and lateral cervical x-ray examination in the Second Affiliated Hospital of Soochow University were included in the study. Age, gender, previous spinal trauma, and disease history of each subject were recorded. After screening, 127 effective samples were finally obtained. When taking lateral cervical radiographs, all subjects placed their neck in a neutral position and looked straight ahead with both eyes. On the obtained lateral x-ray images, a straight line was drawn from the radix to the anterior clinoid process; another line was made along the posterior edge of the C2 vertebral body; and the angle between the two lines was measured, which was defined as the “horizontal view-axial angle.” The angle formed by the tangent of the posterior edge of the C2 vertebra and C7 vertebral body is the “C2–C7 angle,” which was used to describe the curvature of the lower cervical vertebra. The normal range of horizontal view-axial angle and its relationship with C2–7 angle were evaluated.

**Results:**

The average C2–C7 angle of male subjects was (14.0° ± 7.4°), while that of female subjects was (11.09° ± 7.36°). The average horizontal view-axial angle of male subjects was (92.79° ± 4.52°), and that of female subjects was (94.29° ± 4.50°). Pearson correlation test showed that there was a significant negative correlation between horizontal view-axis angle and C2–C7 angle.

**Conclusions:**

For patients with atlantoaxial instability diseases, the horizontal view-axis angle is expected to be a sagittal parameter to guide the intraoperative reduction and evaluate postoperative outcomes.

## Introduction

Craniocervical junction (CVJ) is a tubular area surrounded by the base of the occipital bone, atlas, axis, and ligaments surrounding the foramen magnum, covering the medulla oblongata and the upper spinal cord. The deformities of this region, such as atlantoaxial dislocation (AAD), basilar invagination (BI), and some other diseases may affect the stability of CVJ. The loss of stability between atlas and axis allows the dislocated vertebrae to move horizontally and vertically, leading to the compression of the brainstem and cervical spinal cord, thus resulting in a series of clinical symptoms.

Patients with atlantoaxial instability always require surgical treatment. Many scholars have reported numerous surgical methods (1–5), and the common purposes of these methods are reduction, decompression, fixation, and fusion. The effect of decompression of the medulla oblongata and cervical spinal cord directly affects the improvement of postoperative neurological symptoms, while appropriate reduction and internal fixation is the key to restore and maintain the stability of the CVJ and cervical spine (6–8).

Insufficient and excessive intraoperative reduction both increase the risk of postoperative complications and easily lead to serious adverse consequences, which puts forward requirements for accurate reduction. At present, academic studies on atlantoaxial joint instability diseases are mainly focused on how to achieve more accurate reduction during surgery. Restoration of the normal sagittal arrangement of the craniocervical junction and maintenance of its sagittal balance are considered to be an important factor affecting the postoperative clinical effect (3, 8–10). In recent years, several different sagittal measurement parameters have been reported by many scholars to guide intraoperative reduction and to evaluate the effect of surgical decompression and reduction. However, there is still no widely recognized, simple, and easily measured parameter that can be directly used to guide intraoperative reduction.

Based on the measurement of lateral radiography of cervical spine in asymptomatic subjects, we proposed a new parameter to further describe the normal alignment of craniocervical junction. It is expected to be used to guide the reduction of CVJ during operation.

## Methods

A total of 159 asymptomatic subjects between 18 and 65 years of age who underwent physical examination in Second Affiliated Hospital of Soochow University from November 2018 to June 2020 were included in the study. Each subject's name, sex, and age were recorded, and previous spine-related illnesses and injuries were asked. Samples were screened according to the following inclusion and exclusion criteria, and a total of 127 effective samples were obtained, including 58 males and 69 females. Our study was approved by the hospital ethics committee.

**Inclusion criteria:** (1) The age range ranged from 18 to 65 years. (2) No history of spinal disease or trauma. (3) Radiographs showed no obvious lateral curvature, vertebral fusion, dislocation, and other abnormalities of the cervical spine. (4) T1 slope was measured in the range of 13°–25° (21–23).

**Exclusion criteria:** (1) Less than 18 years old or more than 65 years old. (2) Have a history of spine-related diseases and/or trauma. (3) Radiographs showed that there were abnormalities such as scoliosis, vertebral fusion, and dislocation of cervical vertebra. (4) The slope of T1 vertebral body was measured as less than 13° or more than 25°. (5) Unable to stand upright or the height of the shoulders is not equal when standing upright.

All subjects enrolled in this study underwent anteroposterior and lateral x-ray examination of cervical spine. When subjects were taking lateral cervical spine x-ray, kept subjects standing upright, made sure that their shoulders are at the same height, asked them to look forward with both eyes, kept their neck in a neutral position. The lateral cranial–cervical x-ray images were obtained by adjusting the scope of the images so that the nasal, occipital could be captured.

**Figure 1:**
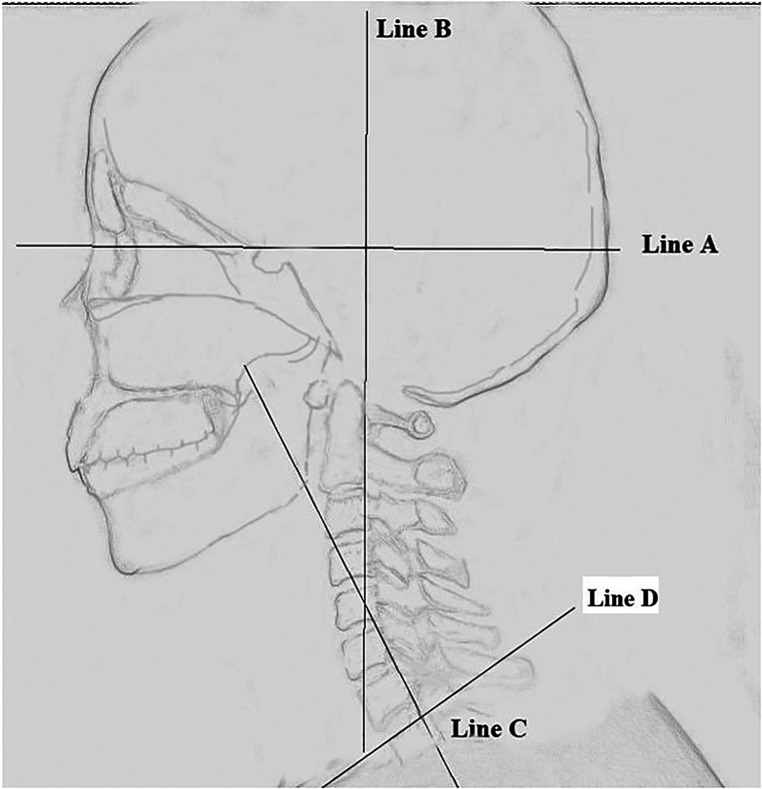
Schematic diagram of horizontal view-axis angle and C2–C7 angle. Line A was made from the radix of the nasal bone to the anterior clinoid process. Line B was made along the posterior edge of the C2 vertebral body. Line C was made along the posterior edge of the C7 vertebral body. Horizontal view-axis angle. The angle between Line A and Line B. C2–C7 angle, The angle between Line B and Line C.

On the lateral radiograph, four lines were made: (1) Line A was made from the radix (Figure 1) to the anterior clinoid process. (2) Line B was made along the posterior edge of the C2 vertebral body. (3) Line C was made along the posterior edge of the C7 vertebral body. (4) Line D was made along the upper edge of T1 vertebral body.

**Figure 2:**
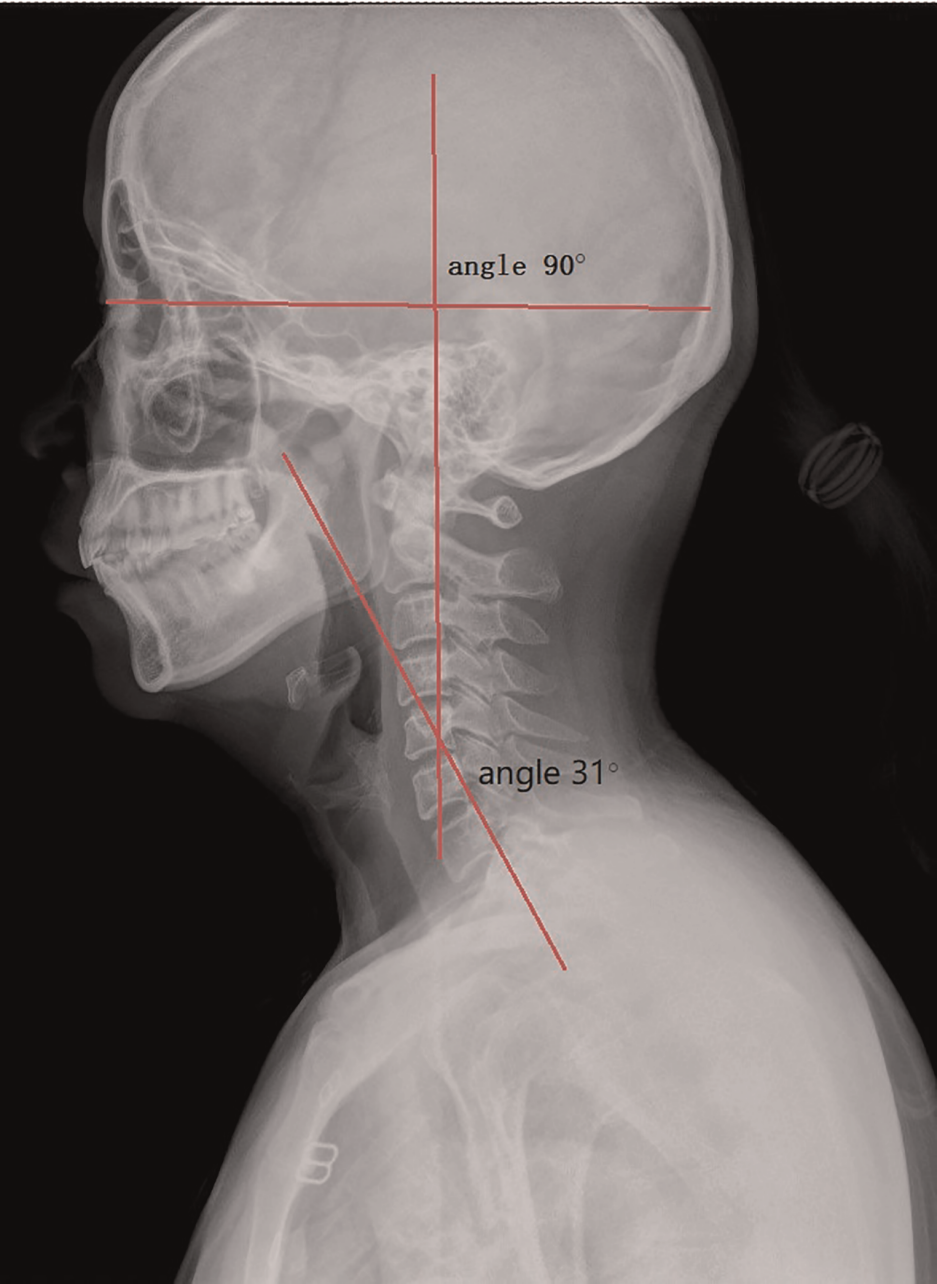
Angles measured on the neutral lateral x-ray.

**Figure 3:**
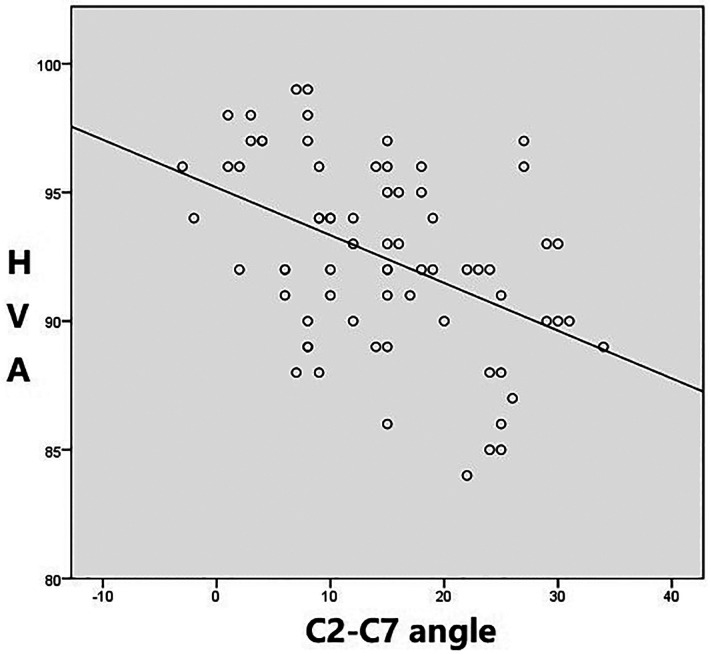
Negative correlation between horizontal view-axis angles and C2–7 angles. (Male group *r*=−0.55 [*p* < 0.01]).

Three angles were measured: (1) The angle between Line A and Line B was defined as “horizontal view-axis angle” (HVA). (2) The angle between Lines B and C was “C2–C7 Angle”. (3) The angle between Line D and the horizontal reference line is “T1 slope” (Figures 2, 3).

Three spine surgeons, in fellowship training, independently measured the three angles to assess accuracy and reliability, using validated software (Neusoft PACS/RIS). Each doctor measured the three angles repeatedly, at least 2 days apart.

## Statistical analysis

The distances and angles between related structures were measured using digital display Vernier calipers and a digital display angle scale. The results are expressed as the mean ± standard deviation (*SD*). Using Student's *t*-tests, statistical analyses were performed (SPSS for Windows, Version 21.0; IBM Corporation, Armonk, NY, United States), with significance set at a *p*-value of < 0.05.

## Results

Of the 127 selected individuals, the mean horizontal view-axis angle was (94.29° ± 4.50°) in female and (92.79° ± 4.52°) in male, it was not statistically significant (*p* > 0.05). The mean C2–C7 angle was (11.09° ± 7.36°) in female, while in male, was (14.0° ± 7.40°), it was statistically significant (*p* < 0.05).

**Figure 4:**
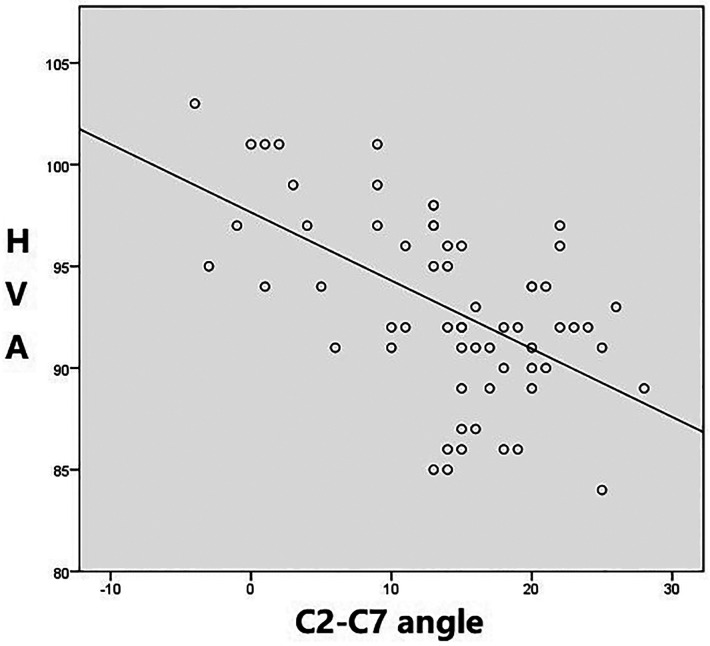
Negative correlation between horizontal view-axis angles and C2–7 angles. (Female group *r*=−0.79 [*p* < 0.01]).

**Figure 5:**
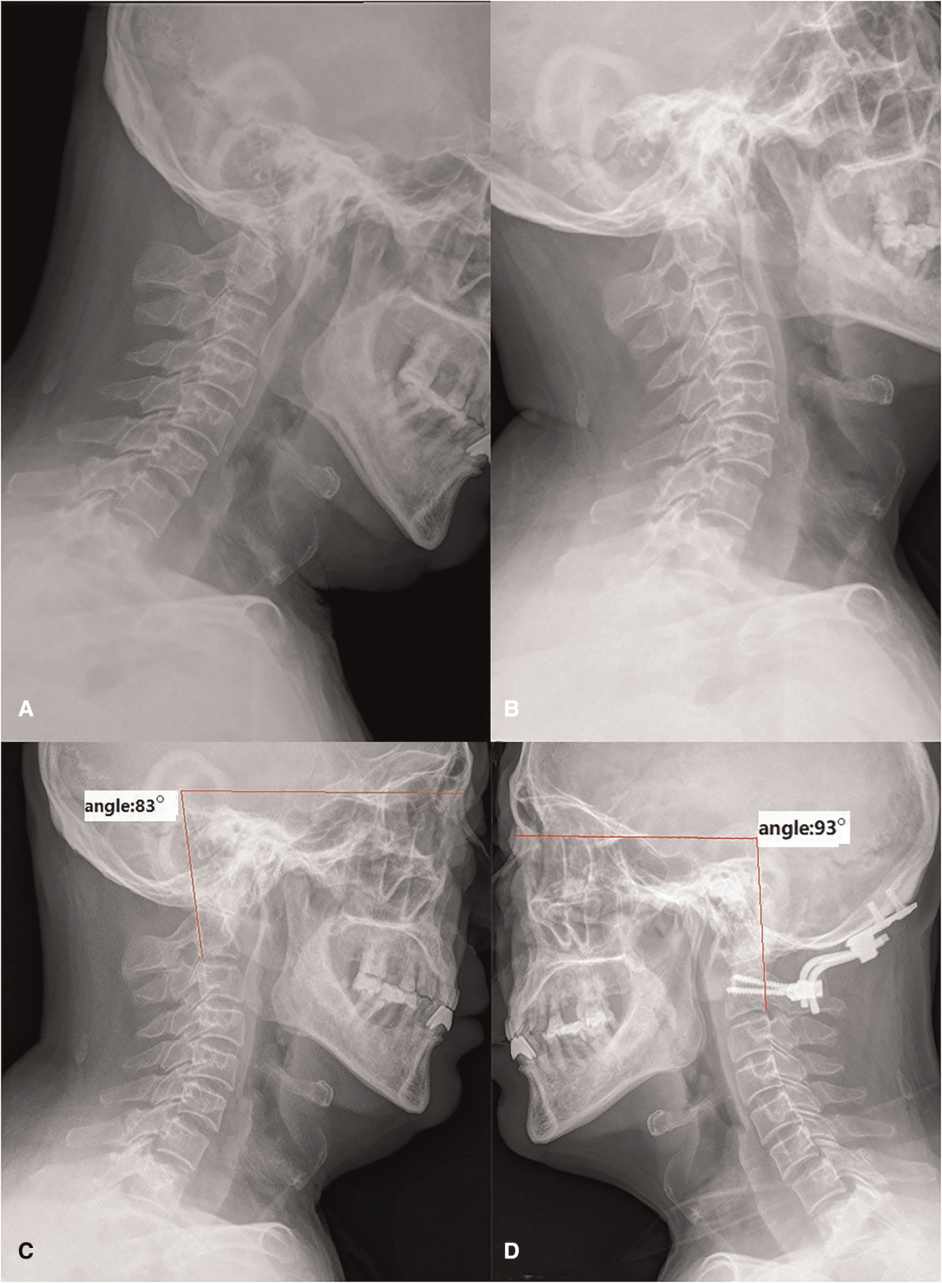
A case of a 57-year-old male patient with BI and AAD. A 57-year-old male, preoperative flexion-extension radiograph showed the atlantodental interval (ADI) was enlarged and the dislocated odontoid process invaginated into the foramen magnum (A,B). The lateral radiograph with the neck in a neutral position showed the HVA was 83° before surgery (C). The angle was reduced to 93° after operation (D). BI, basilar invagination; AAD, atlantoaxial dislocation; HVA, horizontal view-axis angle.

Pearson analysis was used to analyze the relationship between HVA and C2–C7 angle. In both male and female subjects, a significant negative correlation was observed between the two angles, that is, with the increase of the heads-axial angle, the angle of C2–C7 angle decreased, and vice versa. The correlation coefficient between the HVA and C2–7 angles was *r* = −0.79 (*p* < 0.01) in female and *r* = −0.55 (*p* < 0.01) in male (Figures 4, 5).

## Discussion

The purpose of surgical treatment of atlantoaxial instability is to relieve the compression of brainstem and spinal cord, restore the normal sagittal alignment of cervical spine, and maintain its long-term stability. The sagittal alignment of cervical spine is considered to obtain and maintain horizontal gaze. Realignment and maintaining the sagittal balance of the cervical spine is considered to be an important factor affecting the postoperative clinical results. Insufficient or excessive reduction during the operation will affect the surgical effect, increasing the risk of postoperative complications (8–12). How to achieve accurate reduction is the main research direction for the treatment. Different scholars have reported several sagittal parameters to guide surgery (11–20, 24, 25) such as OC–C2 angle, cervico-medullary angle (CMA), clivus-canal angle (CCA), Frankfort-axial angle (FXA), and so on.

Studies of the OC–C2 angle have revealed that the curvature of the upper cervical spine affects the curvature of the lower cervical spine. There is a negative correlation between the two angles, when the lordosis of OC–C2 angle (represents the curvature of the upper cervical spine) increases, C2–C7 angle (represents the curvature of the lower cervical spine) will become more kyphotic, and vice versa. This reminds surgeons to pay attention to the reduction and fixation of the upper cervical curvature during the operation (11–14). Too large or too small upper cervical curvature will lead to compensatory abnormal curvature of the lower cervical spine (15), which will increase the risk of postoperative complications. However, considering that the structures such as occipital and clivus are often malformed in patients with atlantoaxial instability, it is doubtful whether the measured curvature can play a guiding role in reduction surgery. Cervico-medullary angle and clivus-canal angle (15–19) can directly or indirectly reflect the compression of brainstem and cervical spinal cord, which plays an important role in preoperative planning and postoperative efficacy evaluation for patients with atlantoaxial instability. However, the CMA could not be measured on digital radiography (DR) images, the measurement of the CCA is also affected by bone malformations at the craniocervical junction, and the standard for correction of the CCA during surgery has not been determined. In a recent study, Liu et al. first used the horizontal line of sight to evaluate sagittal reduction in patients with atlantoaxial instability (20). They regarded the Frankfort horizontal line (FH, a line connecting the external auditory canal and the inferior margin of the orbit) as the line of sight, and first introduced FXA (the angle between FH, and tangent line of posterior edge of axial). But the position of external auditory canal cannot be clearly displayed on DR images, which will make it difficult to measure the FH line. On sagittal CT images, the measurement of FH line is also complicated and often requires multiplanar reconstruction of CT data to determine. The FH line may not be suitable for intraoperative and postoperative evaluation.

Inspired by the study of Liu et al., we introduced the concept of “horizontal line” into our study, and established a new measurement parameter “horizontal view—axis angle” on craniocervical DR images. When the subject is upright, the neck is in a neutral position and both eyes are looking forward, the radix is close to the midpoint of the line between the two eyeballs. On the lateral x-ray images, we believe that this point may be approximately regarded as the convergence point of external visual information into the eyes. Visual information received by the retina, through the optic nerve into the skull and then to the brain. Optic walks through optic canal into the skull, while the anatomic position of the anterior clinoid process is close to the optic canal. Therefore, on lateral cranial–cervical x-ray images, the line between the radix and the anterior process can be approximately regarded as the path of visual information convergent from the outside to the eyes and further transferred to the inside of the brain, so we set the line between these two points as our horizontal line of sight, the angle between this line(Line A) and the tangent line of posterior margin of C2 (Line B) is defined as the “horizontal view-axis angle,” this is an angle that reflects the relationship between the skull and the upper cervical spine. In this study, we observed a statistically significant negative correlation between the HVA and C2–C7 angle, which means the change of horizontal view-axis angle may influence the curvature of subaxial cervical spine. For patients with atlantoaxial instability, after surgical reduction and fixation of the angle between the occipital and axis, the HVA is also fixed. The subaxial cervical spine plays a major compensatory role to obtain horizontal gaze (15). Therefore, the reduction of the HVA during the operation is particularly important, which will affect the risk of postoperative complications.

**Figure 6:**
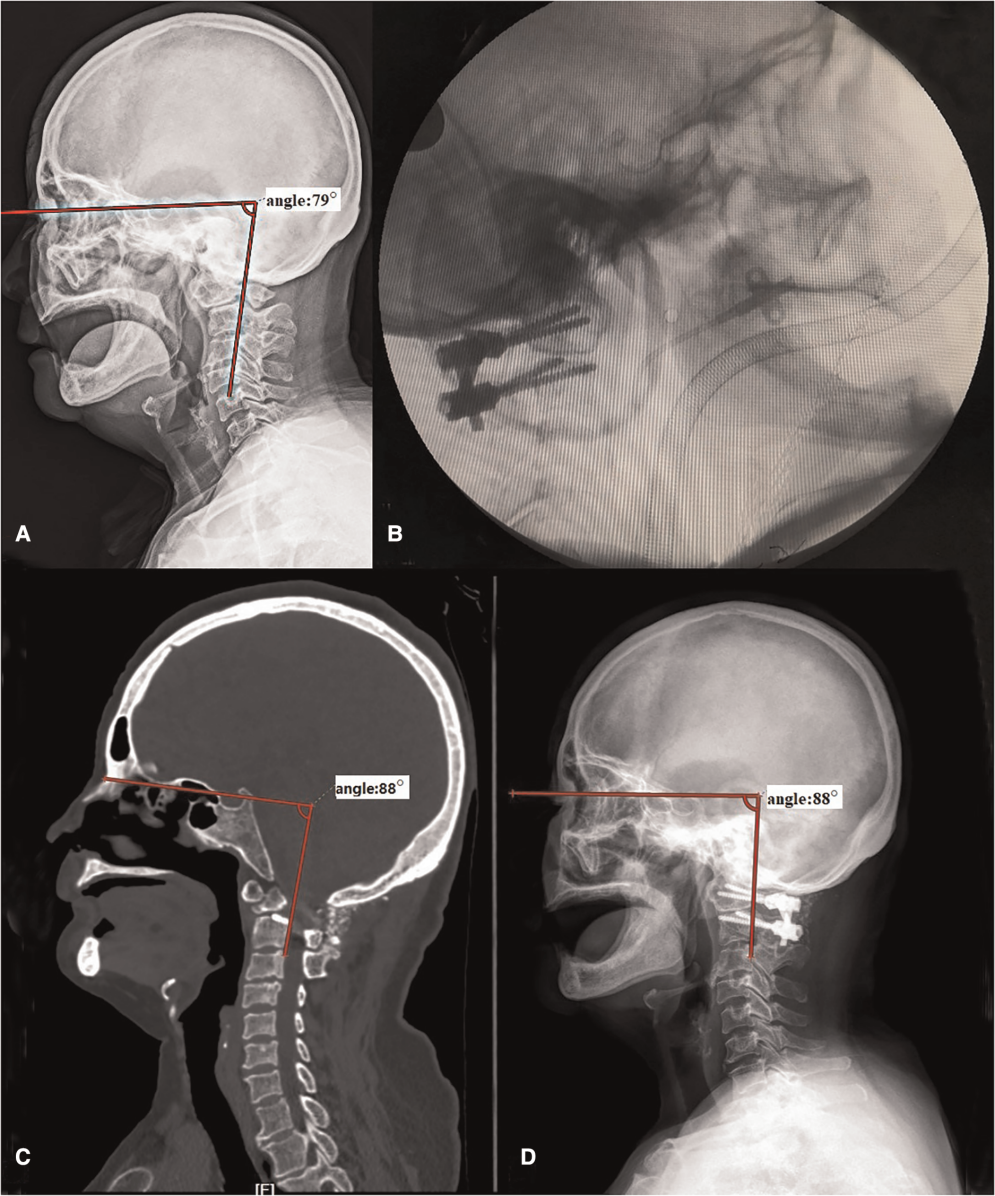
A case of a 69-year-old female patient with Os odontoideum. Before operation, the HVA was 79° (A). With the help of C-arm, we kept the HVA in the normal range during surgery (B). The HVA measured on x-ray (C) and CT (D) was both 88° after operation. HVA, horizontal view-axis angle.

Because Lines A and B can be clearly showed on intra operative x-ray images and not affected by the abnormal bony structure of CVJ, the HVA can be accurate adjusted during surgery. We can keep the angle in the normal range by traction; then internal fixation can be performed on this premise, which plays a role of guiding reduction during the operation. We show two cases in the figures below (Figure 5, 6, Table 1).

**Table 1:** Clinical data on patients

Figure	Gender	Age	Diagnosis	Preoperative JOA scores	Preoperative HVA	Postoperative JOA scores (3 months)	Postoperative HVA
Figure 5	Male	57	BI and AAD	19	83°	23	93°
Figure 6	Female	69	Os odontoideum	16	79°	20	88°

BI, basilar invagination; AAD, atlantoaxial dislocation; HVA, horizontal view-axis angle; JOA, Japanese orthopedic association.

The HVA is also easy to measure and evaluate on postoperative CT and MRI images, so that the other measurement parameters, such as the CMA or CCA, can be obtained at the same time, which is helpful for the evaluation of postoperative efficacy of patients, and provides a new channel for the further study of craniocervical junction deformity.

Of course, the study still has some limits. Due to the lack of sufficient patients with atlantoaxial instability, the significance of HV A in clinical diagnosis and treatment still needs further research and discussion. It is hoped that more patient data can be collected for relevant evaluation and analysis in subsequent studies.

## Conclusions

In this study, we proposed a new parameter “horizontal view-axis angle,” which can be easily measured on x-ray and CT images and is negatively correlated with the curvature of the lower cervical spine.

During the operation, the angle can be measured clearly by using the C-arm machine, which provides the possibility for accurate reduction during surgery. This angle can also be used in postoperative evaluation.

Based on our current use of this parameter, we think that this “horizontal view-axis angle” can help surgeons to further understand the anatomy of CVJ and be used as an intraoperative and postoperative monitoring index of atlantoaxial instability.

## Data Availability

The raw data supporting the conclusions of this article will be made available by the authors, without undue reservation.
